# The Level of the Transcription Factor Pax6 Is Essential for Controlling the Balance between Neural Stem Cell Self-Renewal and Neurogenesis

**DOI:** 10.1371/journal.pgen.1000511

**Published:** 2009-06-12

**Authors:** Stephen N. Sansom, Dean S. Griffiths, Andrea Faedo, Dirk-Jan Kleinjan, Youlin Ruan, James Smith, Veronica van Heyningen, John L. Rubenstein, Frederick J. Livesey

**Affiliations:** 1 Gurdon Institute and Department of Biochemistry, University of Cambridge, Cambridge, United Kingdom; 2 Nina Ireland Laboratory of Developmental Neurobiology, Department of Psychiatry, University of California at San Francisco, San Francisco, California, United States of America; 3 Medical Research Council Human Genetics Unit, Western General Hospital, Edinburgh, United Kingdom; Albert Einstein College of Medicine, United States of America

## Abstract

Neural stem cell self-renewal, neurogenesis, and cell fate determination are processes that control the generation of specific classes of neurons at the correct place and time. The transcription factor Pax6 is essential for neural stem cell proliferation, multipotency, and neurogenesis in many regions of the central nervous system, including the cerebral cortex. We used Pax6 as an entry point to define the cellular networks controlling neural stem cell self-renewal and neurogenesis in stem cells of the developing mouse cerebral cortex. We identified the genomic binding locations of Pax6 in neocortical stem cells during normal development and ascertained the functional significance of genes that we found to be regulated by Pax6, finding that Pax6 positively and directly regulates cohorts of genes that promote neural stem cell self-renewal, basal progenitor cell genesis, and neurogenesis. Notably, we defined a core network regulating neocortical stem cell decision-making in which Pax6 interacts with three other regulators of neurogenesis, Neurog2, Ascl1, and Hes1. Analyses of the biological function of Pax6 in neural stem cells through phenotypic analyses of Pax6 gain- and loss-of-function mutant cortices demonstrated that the Pax6-regulated networks operating in neural stem cells are highly dosage sensitive. Increasing Pax6 levels drives the system towards neurogenesis and basal progenitor cell genesis by increasing expression of a cohort of basal progenitor cell determinants, including the key transcription factor Eomes/Tbr2, and thus towards neurogenesis at the expense of self-renewal. Removing Pax6 reduces cortical stem cell self-renewal by decreasing expression of key cell cycle regulators, resulting in excess early neurogenesis. We find that the relative levels of Pax6, Hes1, and Neurog2 are key determinants of a dynamic network that controls whether neural stem cells self-renew, generate cortical neurons, or generate basal progenitor cells, a mechanism that has marked parallels with the transcriptional control of embryonic stem cell self-renewal.

## Author Summary

Neural stem cells make all of the neurons in the brain. A key feature of these cells is the ability to regulate the balance between making more neural stem cells, the process of self-renewal, and making nerve cells, the process of neurogenesis. Too much self-renewal would result in a brain with too few neurons and abnormal circuitry; too much neurogenesis would deplete all of the neural stem cells too quickly, resulting in a small brain and neurological abnormalities. Little is currently known of the how neural stem cells control this fundamental choice. We used one transcription factor, Pax6, which is important for this decision, as an entry point to define the cellular networks controlling neural stem cell self-renewal and neurogenesis in the developing mouse brain. We found that the relative amount of Pax6 controls the balance between self-renewal and neurogenesis in neural stem cells. Increasing Pax6 levels drives the system towards neurogenesis, at the expense of self-renewal, by turning on a genetic programme for making neurons, whereas decreasing Pax6 turns off the genetic programme for neural stem cell self-renewal. In both cases, altering the levels of Pax6 ultimately leads to a small brain, but through very different mechanisms.

## Introduction

A fundamental feature of neural development is the production of defined types of neurons in a temporal order from multipotent, regionally-specified neural stem and progenitor cells [1]. During nervous system development, maintaining the balance between stem cell self-renewal and neurogenesis is essential for the generation of the correct proportions of different classes of neurons and subsequent circuit assembly. Little is known of the molecular control of the key neural stem (NS) cell properties of multipotency and self-renewal. This is in contrast to other classes of stem cells, most notably embryonic stem (ES) cells, in which a group of three transcription factors, Sox2 and the two ES-specific factors Oct4 and Nanog, co-operate to control pluripotency and self-renewal in a non-redundant manner [2],[3].

The paired-domain, homeodomain-containing transcription factor Pax6 is highly conserved among vertebrate and invertebrate species and is essential for the development of much of the central nervous system, including the eye, spinal cord and cerebral cortex, as well as pancreatic islet cells [4]–[7]. Detailed analyses of neocortical and retinal development in mice mutant for Pax6 have identified defects in neural stem and progenitor cell proliferation, multipotency, neurogenesis, the generation of specific types of neurons, and marked changes in spatial pattern [8]–[19]. In the neocortex, loss of Pax6 function results in microcephaly, abnormal development of the secondary progenitor population of the subventricular zone (SVZ, also known as basal progenitor cells, BP cells) and a disproportionate reduction in the production of later-born, upper layer neurons [12], [14], [15], [17], [20]–[23].

Given the functions of Pax6 in stem cell self-renewal/proliferation and neurogenesis, a potentially fruitful approach to uncovering cellular pathways controlling these processes is to identify the downstream targets of Pax6 in neocortical stem cells. Therefore, in this study we used Pax6 as an entry point to define the cellular networks regulating neural stem cell self-renewal and neurogenesis by combining chromatin immunoprecipitation (ChIP) to identify Pax6-bound promoters with the anatomical phenotypes and gene expression changes observed in Pax6 mutant cortices.

To do so, we identified where in the genome Pax6 binds in mouse neocortical stem cells during normal development, defining the potential cellular networks regulated by Pax6. As binding does not necessarily imply regulation, we also studied the transcriptional consequences of altering Pax6 levels in the neocortex *in vivo*. Developing tissues are sensitive to Pax6 dosage: heterozygous human mutations in Pax6 result in aniridia and forebrain abnormalities [24],[25], as do a number of mouse mutations (for reviews, see [7],[26]), humans and mice homozygous for Pax6 mutations typically lack eyes and have marked microcephaly and absent olfactory bulbs [5],[6],[19], whereas increasing Pax6 levels in transgenic mice results in microphthalmia and forebrain abnormalities [27]–[29]. Therefore, we examined the transcriptional consequences of both increasing and decreasing Pax6 levels in the developing cortex to identify those promoters actively regulated by Pax6 in neocortical stem and progenitor cells.

We find that Pax6 controls the balance between self-renewal and differentiation in neural stem cells in a dose dependent manner, positively and directly regulating cohorts of genes that promote self-renewal, basal progenitor cell genesis and neurogenesis. In addition, we found that Pax6 interacts with three other regulators of cortical stem cell neurogenesis, Neurog2, Ascl1 and Hes1. The four transcription factors regulate one another and many of the same target genes in an antagonistic manner, defining a core self-renewal/neurogenesis network that is dependent on a critical level of Pax6. In support of this, we found in phenotypic analyses of Pax6 gain and loss of function cortices that an increase in Pax6 levels drives the system towards neurogenesis and basal progenitor cell genesis at the expense of self-renewal, whereas removing Pax6 reduces cortical stem cell self-renewal. In both cases, altering the levels of Pax6 ultimately leads to microcephaly, but through different cellular and biological pathways.

## Results

**Figure 1:**
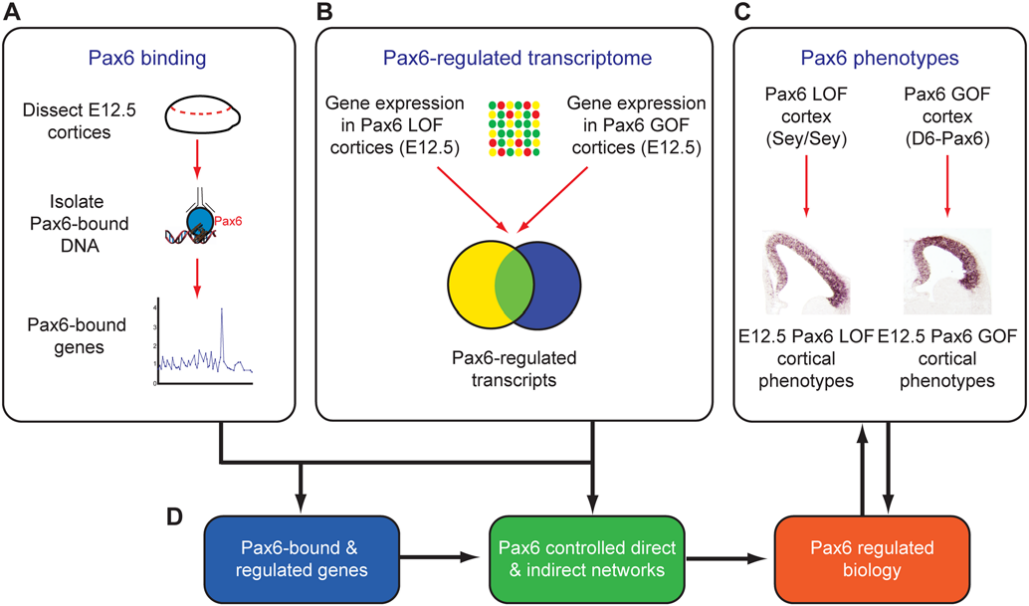
Strategy to characterise the transcriptional networks regulated by Pax6 in neocortical stem cells. (A) Location analysis was performed for Pax6 binding in the genome of neocortical stem and progenitor cells at embryonic day 12.5 (E12.5) to define those promoters bound by Pax6 *in vivo*. (B) To define the transcriptional responses downstream of Pax6, both direct and indirect, global gene expression was compared at E12.5 between wild-type and Pax6 loss-of-function (LOF) cortices (Sey/Sey) and also between wild-type and Pax6 gain-of-function (GOF) cortices (D6-Pax6). (C) Phenotypic analyses of the Pax6 gain- and loss-of-function cortices were used to both interpret the gene expression changes observed upon altering Pax6 dosage and also to test the hypotheses generated from the combination of the Pax6 binding and regulation data (D).

The goal of this research was to define the genetic networks directly and indirectly regulated by Pax6 in neocortical stem and progenitor cells and to characterise the biological networks of which Pax6 is a component. To do so, we combined location analysis of Pax6 bound genomic binding sites in neocortical stem and progenitor cells *in vivo* with transcriptome analysis of the changes in gene expression resulting from altered levels of Pax6, together with phenotypic data from these and previous studies of Pax6 gain- and loss-of-function in the developing mouse cerebral cortex (Figure 1). Finally, we integrated these data with expression data from previous studies of other transcription factors that have roles in controlling neocortical neurogenesis, most notably Neurogenin2 (Neurog2), Hes1 and Ascl1/Mash1 [15],[30],[31], in order to extend our knowledge of the biological networks in which Pax6 operates.

### Identification of Pax6 Target Genes in the Developing Neocortex by Chromatin Immunoprecipitation

To identify promoters targeted by Pax6, we applied *in vivo* location analysis to identify where in the genome Pax6 is bound in neocortical stem and progenitor cells. We carried out chromatin immunoprecipitation (ChIP) for Pax6-bound DNA in the developing mouse neocortex at embryonic day 12.5 (E12.5), at which stage Pax6 is expressed by all neocortical ventricular zone stem and progenitor cells, but not by post-mitotic neurons [32]. A set of three biologically independent Pax6 ChIP experiments was carried out using oligonucleotide microarrays spanning ∼8.5 kb around the transcriptional start sites of over 17,000 mouse genes (see Materials and Methods for details). As the arrays used sample the proximal promoters of these genes, this analysis will not include intronic or distally located enhancers bound by Pax6, such as the Pax6-bound cortical enhancer of Neurog2 expression, found ∼9 kb upstream of the Neurog2 start site [33],[34].

**Figure 2:**
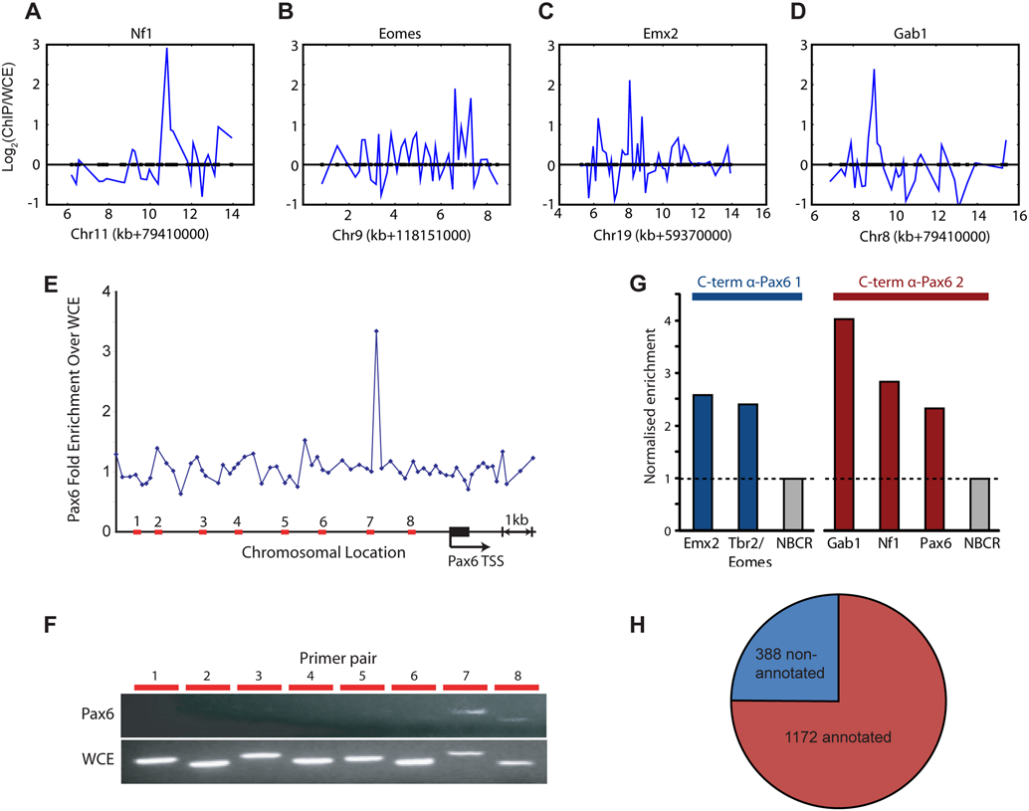
*In vivo* location analysis identifies the set of genes whose promoters are bound by Pax6 in neocortical stem and progenitor cells. (A–D) Identification of Pax6-bound promoters. ChIP array data for four typical Pax6-bound promoters, all of which were calculated as significantly bound above background levels (see Materials and Methods for details). Each graph plots fold-enrichment (in log base 2) on the y-axis, against relative chromosomal position on the x-axis. (E,F) Validation of the specificity and accuracy of the binding of the Pax6 promoter by Pax6, confirmed by qPCR in (G). Scanning PCR primers were designed to amplify DNA from a series of genomic regions in the Pax6 promoter (red bars represent amplified regions in the plot of fold-enrichment, y-axis, against relative chromosomal position, x-axis). The peak of Pax6 binding from the ChIP array analysis is shown in E. Pax6 binding was confirmed in this assay only in the region around the oligonucleotide, as shown in the gel images in F. Pax6, Pax6 ChIP; WCE, whole cell extract. (G) Validation of Pax6-binding to a set of promoters by quantitative PCR in independent ChIPs (not used for the array hybridisations) using two different Pax6 polyclonal antibodies. Enrichment of a region is calculated relative to that observed in control ChIPs without the primary antibody and normalised to a non-bound control region (NBCR). (H) Three independent ChIPs and promoter array hybridisations were carried out for Pax6, in which Pax6-bound material was compared with total genomic DNA (whole cell extract, WCE) to identify regions enriched in the Pax6-ChIP material by raw ratio measurements. Pax6 is found on the promoters of 1560 genes, 1172 (75%) of which are annotated genes (Table S1).

Data from the three experiments were analysed to identify regions of promoters bound by Pax6 in each ChIP and the individual analyses combined to define a set of 1560 genes with Pax6-bound promoters (Figure 2; Table S1), of which 1172 are genes with known or predicted functions (Figure 2H). A set of five genes defined as Pax6-bound by the array study were confirmed as bound by qPCR analysis of additional Pax6 ChIP from the E12.5 cortex using two different Pax6 polyclonal antibodies (Figure 2G).

### Pax6 Directly Binds to Genes that Control Self-Renewal and Neurogenesis

**Figure 3:**
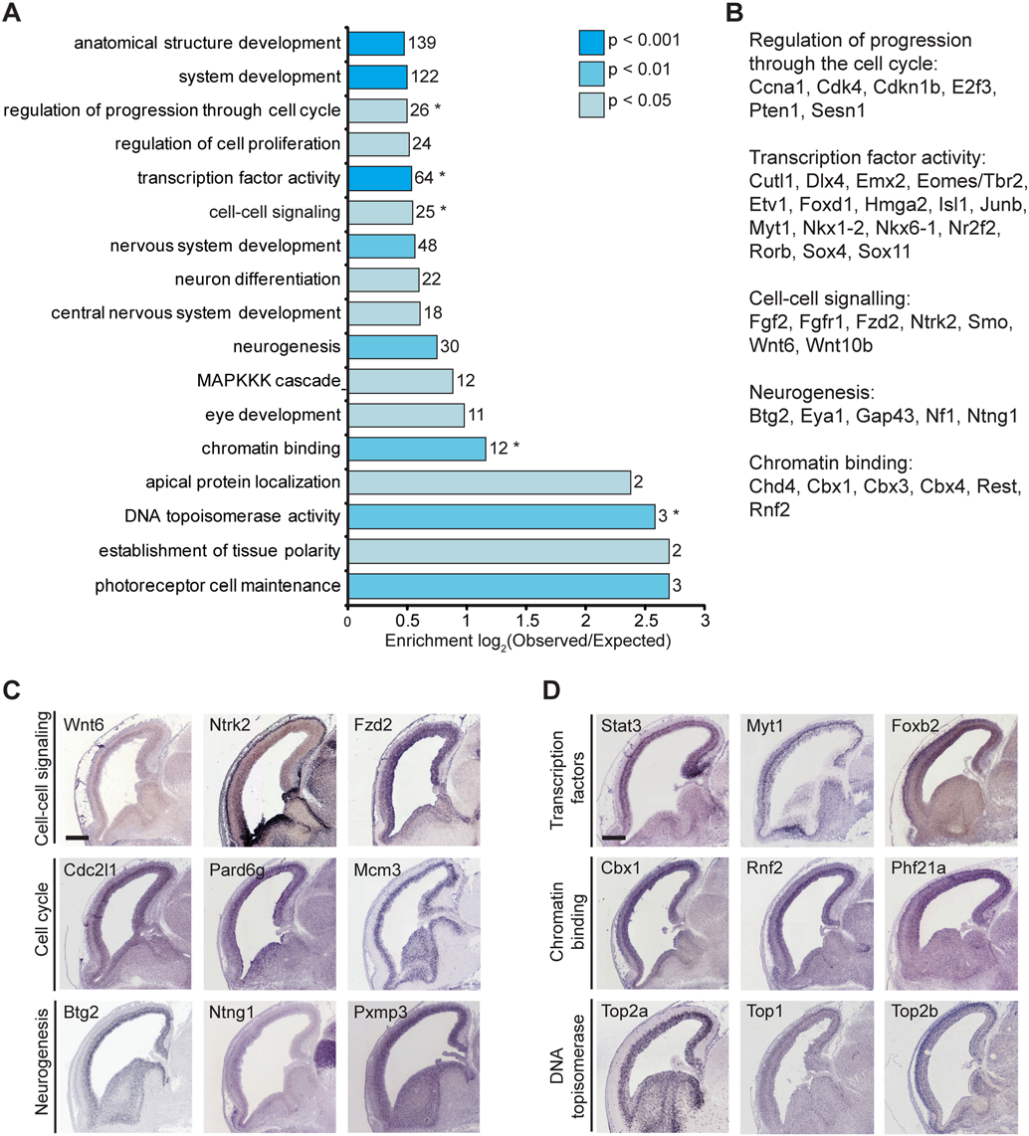
Pax6-bound genes are enriched in genes required for nervous system development. (A) Gene Ontology analysis for enrichment of categories of genes in the Pax6-bound set of genes. The set of Pax6-bound genes was compared to the whole genome to identify functional or biological sets over-represented in the Pax6-bound set, as described in the Materials and Methods. (B) Examples of genes in selected functional categories shown in A. (C,D) Cellular localisation of genes representative of some of the biological (C) and molecular (D) categories shown in (A), at E14.5 from the public *in situ* hybridisation database GenePaint [38]. The majority of genes are expressed in neocortical stem and progenitor cells in the ventricular zone (VZ) and basal progenitor cells in the subventricular zone (SVZ), with a significant minority also expressed in differentiating neurons in the cortical plate (CP). Scale bars, 500 µm.

Functional annotation of the set of Pax6-bound genes by Gene Ontology analysis found a significant enrichment for developmental processes among Pax6 target genes, including nervous system and eye development (Figure 3). Broad functional categories enriched in the Pax6-bound set included cell cycle regulation, regulation of proliferation, cell-cell signalling, neurogenesis and neuron differentiation. At the functional level, Pax6-bound genes are enriched for genes with transcription factor activity and chromatin binding (Figure 3A and 3D). Pax6 binds transcription factors that are expressed in stem and progenitor cells (Pax6 itself, Hmga2, Cutl1, Nr2f2, Emx2, Sox9, Neurog3, Tle1) and basal progenitor cells in the cortex (Eomes/Tbr2, Neurod1), as well as to transcription factors expressed in newly born, differentiating neurons (Sox4, Sox11) or in terminally differentiating cortical neurons (Rorb, Etv1). Together with the binding of Pax6 to sets of regulators of cell cycle progression and proliferation (for example, Ccna1, Pten, Cdkn1b, Cdk4, Fzr1 and Ctnnb1), regulation of these transcription factors confers the potential for Pax6 to control stem cell self-renewal/proliferation, neurogenesis and cell fate determination within the cortex.

### Transcriptome Analysis Following Changes in Pax6 Levels *In Vivo*

**Figure 4:**
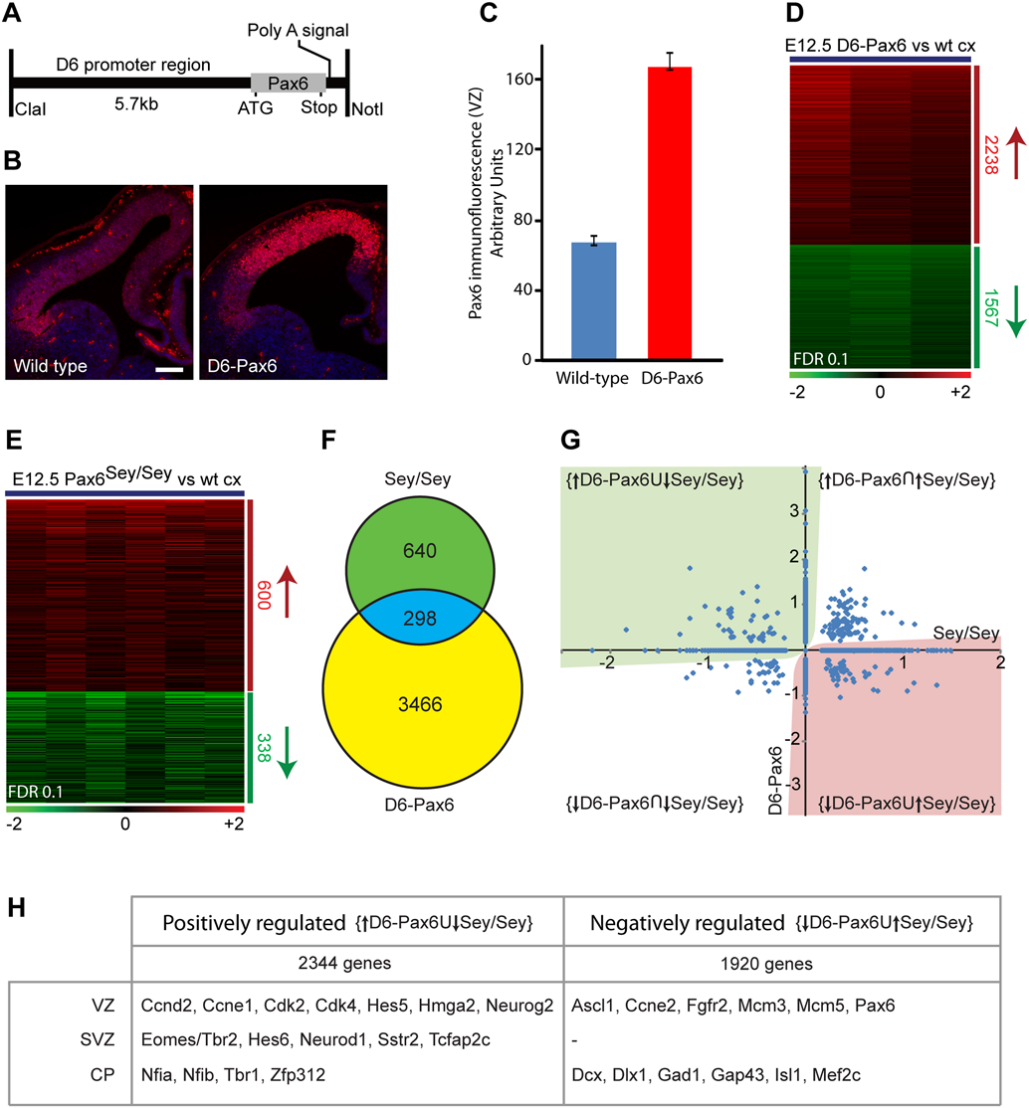
Expression analysis of Pax6 gain and loss-of-function cortices identifies sets of genes dependent on Pax6 for expression. (A) Generation of transgenic mice over-expressing Pax6 specifically in the developing cerebral cortex, using the D6 promoter [35]. (B) Fluorescent immunohistochemistry for Pax6 in control and D6-Pax6 E12.5 cortices demonstrates the marked increase in Pax6 protein in the transgenic cortex. Scale bar, 100 µm. (C) Quantification of the intensity of anti-Pax6 immunofluorescence in WT and D6-Pax6 cortical ventricular zones, relative to signal in the basal ganglia in which the D6 transgene is not expressed. Error bars, s.e.m. (D) Microarray analysis of gene expression changes in the cortex of D6-Pax6 mice at E12.5. Gene expression in cortices from single E12.5 embryos was compared between mice carrying the D6-Pax6 transgene and control littermates in a set of 6 paired, dye-swapped array hybridisations. Paired hybridisations were averaged and genes showing reproducible, statistically significant (FDR<0.1) changes in expression were clustered to identify 1567 up-regulated and 2238 down-regulated transcripts (see Table S2 for details). (E) Microarray analysis of gene expression changes in the cortex of Pax6 mutant (Sey/Sey) mice at E12.5. Gene expression in cortices from single E12.5 embryos was compared between Sey/Sey mice and control littermates in a set of 6 dye-swapped array hybridisations. A set of 938 genes showing reproducible, statistically significant (FDR<0.1) changes in expression were clustered to identify 338 down-regulated and 600 up-regulated transcripts (see Table S3 for details). (F) A set of 298 genes was found to have altered expression in both the Sey/Sey and the D6-Pax6 E12.5 cortex to a statistically significant level. (G) From the total set of 4404 genes showing altered expression in one of more of the Sey/Sey and D6-Pax6 E12.5 cortices, the union of the sets of genes upregulated in the D6-Pax6 cortex and genes down-regulated in the Sey/Sey cortex defines a set of genes that are positively regulated by Pax6 (both directly and indirectly), denoted by the green shading. In contrast, the union of the sets of genes downregulated in the D6-Pax6 cortex and genes up-regulated in the Sey/Sey cortex defines a set of genes that are negatively regulated by Pax6 (both directly and indirectly), denoted by the red shading. (H) Examples of the genes found in each of the four categories defined in (C).

Pax6 binding data defines the potential networks controlled by Pax6 in this cell type. However, binding does not necessarily equate with activity. Therefore, to characterize the transcriptional networks within which Pax6 acts, we studied gene expression in the E12.5 mouse cortex, using microarrays, following Pax6 gain and loss of function (Figure 4). Intersection of genes that are positively or negatively regulated by Pax6 defined the transcriptional networks dependent on Pax6, without discriminating between direct and indirect regulation.

For Pax6 gain of function, we used the D6 enhancer that drives cortex-specific expression in the ventricular zone and cortical plate [35]–[37] to generate transgenic mice expressing the canonical Pax6 isoform, giving rise to animals with a two-fold increase in Pax6 protein in the ventricular zone, as assessed by immunohistochemistry (Figure 4A–4C). Expression profiling of cortices from single wild-type and D6-Pax6 transgenic littermates at E12.5 found 3784 genes showing significantly altered expression (false discovery rate, FDR, of 0.1), of which 2238 (59%) were upregulated (Figure 4D; Table S2). In comparison, expression profiling of the Pax6 homozygous mutant cortex (Sey/Sey) at E12.5 identified 938 genes with significantly altered expression, of which 600 (64%) were up-regulated (Figure 4E; Table S3). Of the 938 genes showing altered expression in the Pax6 null mutant cortex, 298 (32%) were also altered in expression in the D6-Pax6 cortex (Figure 4F). From the transcriptome analyses, we defined two sets of genes showing Pax6-dependency: the set of genes showing positive dependency on Pax6 was the set formed by the union of genes up-regulated in the D6-Pax6 cortex and the genes down-regulated in the Pax6 mutant cortex (Figure 4F and 4G; 2344 genes); the set of genes showing negative dependency on Pax6 was the set formed by the union of genes down-regulated in the D6-Pax6 cortex and the genes up-regulated in the Pax6 mutant cortex (Figure 4F and 4G; 1920 genes).

Genes demonstrating positive dependency on Pax6 were notable for including many genes expressed in basal progenitor cells, including Eomes/Tbr2, Gadd45g, Neurod1, Tcfap2c and Hes6 (Figure 4H). Genes similarly dependent on Pax6 for expression include genes expressed in cortical stem and progenitor cells associated with self-renewal and proliferation, such as the transcriptional regulators Hmga2 and Hes5, and the cell cycle regulators Cdk2, Cdk4 and Ccne1. However, transcription factors associated with neurogenesis, such as Neurog2, Sox4 and Sox21, also show positive dependency on Pax6 for their expression. In addition, increasing Pax6 expression leads to an increase in expression of transcription factors that are preferentially expressed in cortical neurons of layers 5 and 6 (Tbr1, Zfp312/Fezf2, Nfia, Nfib).

In contrast, genes demonstrating negative dependency on Pax6 included genes with periodic expression in the cell cycle (FoxM1, Mcm3, Mcm5). These genes were reduced in expression in the Pax6-overexpressing cortex, indicating that increasing Pax6 may alter cell cycle length. Together, the transcriptional analyses demonstrate that Pax6 regulates neural stem cell maintenance, basal progenitor cell genesis and neurogenesis.

### Pax6 Bound and Regulated Genes Define the Network Directly Controlled by Pax6 in Neocortical Stem and Progenitor Cells

**Figure 5:**
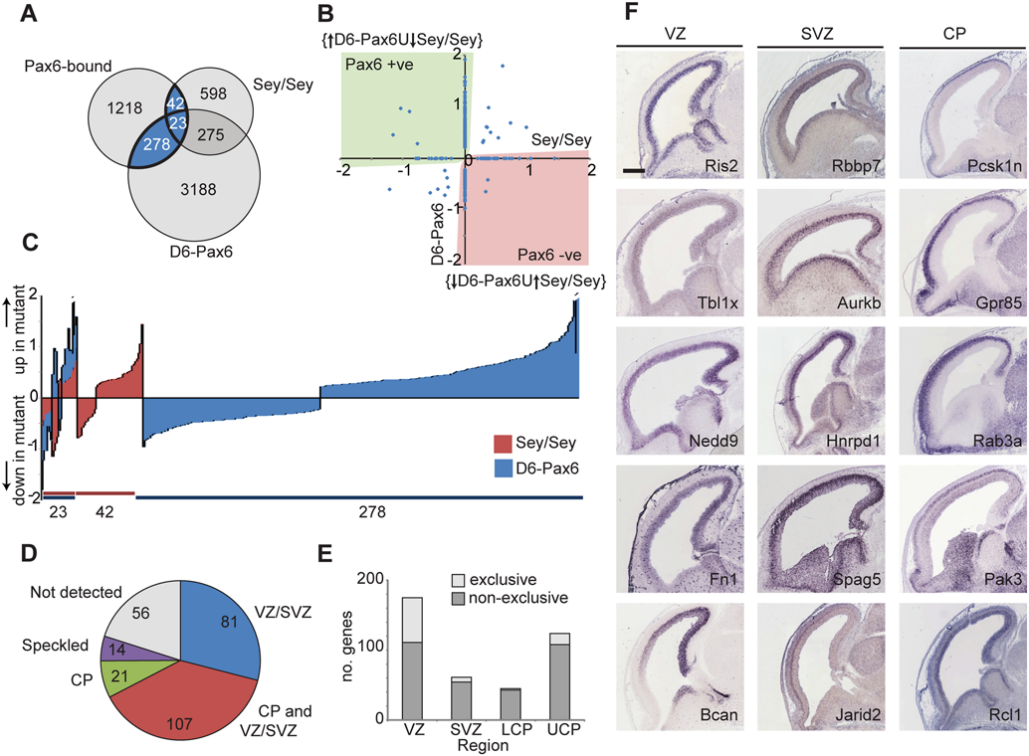
The set of genes bound and regulated by Pax6 in the embryonic cerebral cortex. (A) Identification of a set of 343 genes both bound and regulated by Pax6 by intersecting the three genomics datasets reported here: Pax6 ChIP-on-chip and the array analyses of Pax6 gain- and loss-of-function cortices. The intersection is highlighted in blue (see Table S4 for details). (B) Definition of the sets of genes bound and positively regulated by Pax6 (green shading) and negatively regulated by Pax6 (red shading). (C) Breakdown of the evidence for positive or negative regulation of genes by Pax6: 23 genes show regulation following both gain and loss of Pax6 function, 42 show regulation only upon loss of function and 278 show regulation only upon gain of function. (D–F) In situ hybridisation data for cellular expression of Pax6 bound and regulated genes in whole embryos at E14.5 was available for 279 genes (GenePaint), 56 of which were not detected at this age. Blind scoring of cellular distribution of expression patterns of the bound and regulated genes (D, E) found that the majority of genes were expressed by neocortical stem/progenitor cells (VZ), including substantial numbers of genes expressed in both the VZ and in differentiating neurons, as well as genes exclusively expressed in the VZ (E). Many Pax6 target genes were also expressed by basal progenitor cells in the SVZ. Examples of genes expressed in all three cell types (VZ, SVZ and CP) are shown in F. Scale bar, 500 µm.

To identify those genes that are both bound and regulated by Pax6, we analysed the intersection between the sets of Pax6 bound genes and those genes showing Pax6 dependency from the gain and loss of function transcriptional analyses (Figure 5). Of the set of 1560 Pax6-bound genes identified by the *in vivo* location analysis, 343 (22%) show significantly changed expression in either the Pax6 gain or loss of function cortex at E12.5 (Figure 5A; Table S4). Of the 343 bound and regulated genes, 180 were positively regulated and 143 negatively regulated by Pax6 (Figure 5B and 5C), with 20 genes showing conflicting regulation (either up- or down-regulated upon both gain and loss of Pax6 function).

Using publicly available *in situ* hybridization data (GenePaint.org; [38]), we assigned cellular expression to 279 of the 343 Pax6 bound and regulated genes in the E14.5 mouse cortex, the mRNAs of 223 of which were detectable in the cortex at that age (Figure 5D–5F; Table S5). Ventricular zone expression was found for 188 of those genes (84%), 81 of which were solely expressed in the ventricular and subventricular zone. A noteworthy minority of Pax6 bound and regulated genes (21; 9% of detectable mRNAs) were exclusively expressed in differentiating neurons in the cortical plate.

### Pax6-Regulated Transcriptional and Biological Networks

**Figure 6:**
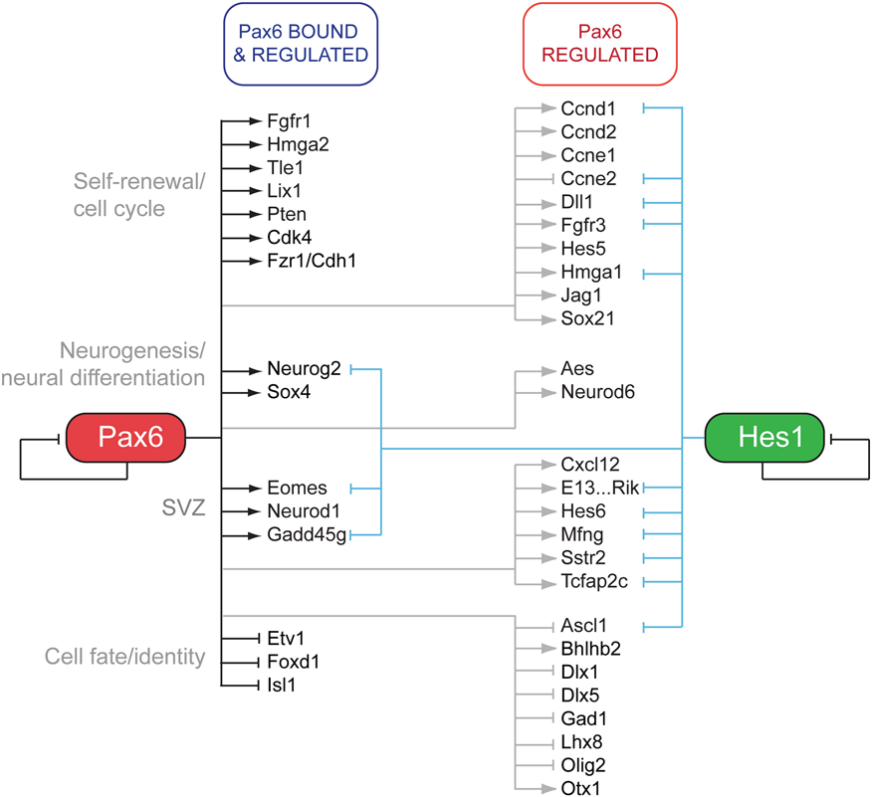
Pax6-regulated transcriptional networks in neocortical stem cells. Pax6 directly regulates sets of genes that control four major processes in cortical neurogenesis: stem cell maintenance/self-renewal, neurogenesis, production of basal progenitor cells and cortical identity (black lines). These direct programmes are underpinned by indirect control of larger numbers of genes involved in each of these processes, as shown by the grey lines. Hes1 suppresses neurogenesis when continuously over-expressed in cortical stem/progenitor cells [51] and that is associated with repression (blue lines) of many of the same genes positively regulated by Pax6, including those genes required for genesis of basal progenitor cells.

The combination of Pax6 binding data with the transcriptional profiling of Pax6 gain- and loss-of-function cortices enabled the delineation of the direct and indirect networks regulated by Pax6 (Figure 6) and prediction of Pax6 functions in cortical stem and progenitor cells. As shown, Pax6 positively controls sets of genes promoting neural stem cell self-renewal and/or maintenance: Pax6 positively regulates transcription of Hmga2, Tle1 and Cdk4 directly, promoting cell cycle progression and neural stem cell maintenance [39]–[41]. Furthermore, Pax6 controls expression of D-class cyclins, Hes5 and Notch ligands indirectly [39],[42],[43], underlining the importance of Pax6 in promoting neural stem cell self-renewal.

However, the self-renewal functions of Pax6 are offset by its binding to, and positive regulation of, genes and transcription factors that promote neurogenesis, including the tumour suppressor genes Pten and Fzr1/Cdh1 and the transcription factors Neurog2 and Sox4 [31], [44]–[46]. In addition, Pax6 directly and positively controls genes that promote basal progenitor cell genesis, including one of the major determinants of the basal progenitor fate, Eomes/Tbr2, as well as Neurod1 and Gadd45g [47]–[49]. Together, these functions of Pax6 indicate that one biological role for Pax6 is to promote neurogenesis by increasing the number of neocortical stem and progenitor cells either exiting the cell cycle or becoming basal progenitor cells, and thus neurons at their next division.

In addition to roles in neurogenesis and self-renewal, Pax6 controls genes that regulate the neocortical identity of the neurons produced in the dorsal pallium. It does so by primarily negatively regulating genes associated with neuronal cell fates, and subpallially-derived inhibitory neuron and interneuron identity in particular, through repression of key transcription factors that confer interneuron fates (Isl1, Foxd1, Ascl1, Dlx1, Lhx8; Figure 6; [50]).

Many of the findings reported here on the functions of Pax6 in neurogenesis and self-renewal are in contrast with the recently described functions of the bHLH transcription factor Hes1, sustained overexpression of which represses many of the genes upregulated by Pax6 [51]. Comparison of the expression data from Hes1 overexpression with the Pax6 data presented here shows that Pax6 and Hes1 have opposing functions on neurogenesis and basal progenitor genesis through the same sets of effector genes (Figure 6): Pax6 positively regulates Neurog2 and Eomes/Tbr2, for example, whereas both of these key transcription factors are repressed by Hes1. As discussed in more detail below, this overlap suggests that Pax6 and Hes1 are both regulating a core mechanism for controlling neural stem cell self-renewal and neurogenesis.

### Pax6 Gain-of-Function Results in an Increase in Neurogenesis and Production of Basal Progenitor Cells

**Figure 7:**
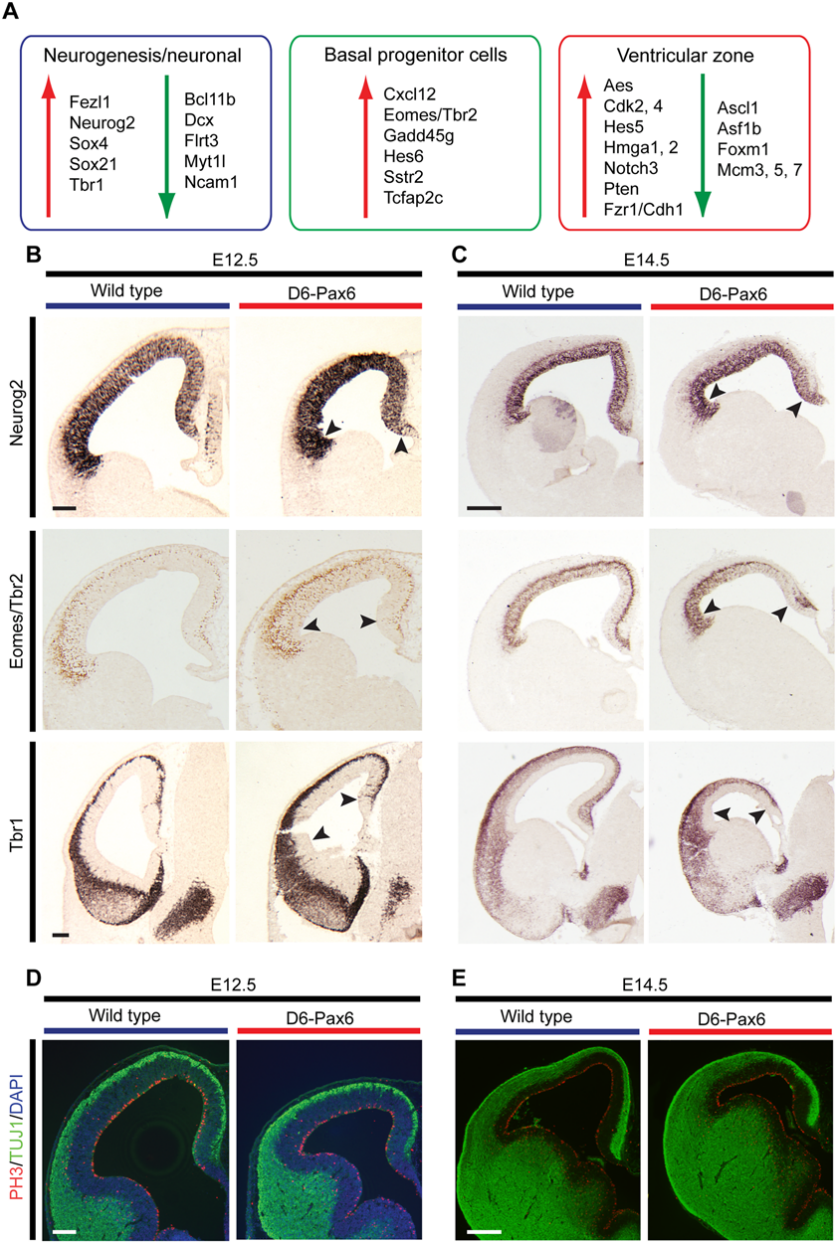
Increased expression of Pax6 specifically in the developing cortex alters neocortical stem cell self-renewal and cell fate determination. (A) Genes characteristic of the three main cell types in the embryonic cortex (stem/progenitor cells in the ventricular zone; basal progenitor cells and differentiating neurons) are changed in expression in the D6-Pax6 cortex: there is a striking increase in a set of genes specifically expressed in basal progenitor cells (Eomes/Tbr2, Hes6, Tcfap2c), indicating that there is an increased production of this cell type when Pax6 levels are increased. Genes specifically expressed in layer 5 and 6 neurons are upregulated when Pax6 levels are increased, as are genes associated with cortical neurogenesis (Neurog2, Sox4, Sox21). Conflicting changes in the expression of cell cycle regulators are observed, with increases in expression of positive regulators of G1 progression (Cdk2, Cdk4, Hmga2) and negative regulators of proliferation (Pten, Fzr1), accompanied by decreased expression of genes specifically transcribed in S- and M-phase (Foxm1, Mcm3, 5 & 7). (B) Over-production of basal progenitor cells (Eomes/Tbr2) and layer 6 neurons (Tbr1) observed at E12.5, resulting from increased Pax6 expression that also upregulates Neurog2 expression. Arrowheads indicate the region of Pax6 over-expression within the cortex, which does not extend throughout the entire lateral dimension of the VZ. Scale bars, 100 µm. (C) The increased expression of Pax6 in the D6-Pax6 cortex results in microcephaly at E14.5 that is more pronounced caudally (where the D6 transgene drives the highest increase in Pax6 expression). The cortical plate is reduced in thickness compared to wildtype, with an overall reduction in the number of neurons (as assessed by Tbr1 staining). Increased Neurog2 expression is maintained at this stage, as is increased Eomes/Tbr2 expression. Scale bar, 200 µm. (D,E) Increased Pax6 expression results in a reduction in total cortical size at E12.5. At both E12.5 and E14.5 the microcephaly is not associated with a marked change in mitotic index, as assayed by phopho-histone H3 staining for cells in M-phase at the ventricular surface. Green, Tuj1; red, phospho-histone H3; blue, DAPI. Scale bars, D, 100 µm; F, 200 µm.

To test the prediction of the functions of the Pax6-regulated network described above, we studied neurogenesis and cell fate determination in the Pax6 gain-of-function cortex (D6-Pax6 transgenic mouse; Figure 7). As predicted, increased Pax6 resulted in a marked increase in the expression of Eomes/Tbr2 at E12.5, demonstrating the increase in the basal progenitor cell population. Furthermore, there was with an increase in early born, layer 6 neurons (Tbr1-expressing cells), without an increase in the total number of neurons produced by this stage of development, compared to controls (as evidenced by the number of cells expressing neuron-specific tubulin, Tuj1, Figure 7).

However, by E14.5 the D6-Pax6 transgenic cortex was significantly smaller than that of controls, with a reduction in total neuron number (Figure 7). The reduction in cortical neuron number at this stage is consistent with the early increase in Pax6 expression driving cortical stem and progenitor cells towards an inappropriately early basal progenitor and neuronal fate. The reduction in cortical size is not due to an increase in cell death in the Pax6 overexpressing cortex, as no significant increase in apoptosis could be detected by TUNEL staining (Figure S1). The early depletion of the stem cell pool reduces the number of stem cells available for neurogenesis at subsequent stages, resulting in an overall reduction in cortical size and total cortical neurogenesis by E14.5 (Figure 7).

### A Cortical Neurogenesis/Self-Renewal Regulatory Circuit Controlled by Pax6

Neurogenin2 (Neurog2) and Ascl1/Mash1 are two proneural bHLH transcription factors with well-established roles in neocortical neurogenesis and cell fate [15],[31]. Both interact with Pax6: Pax6 positively regulates Neurog2 via an enhancer [34], whereas Ascl1 expression is upregulated in Pax6-null cortices [52]. Gene expression profiling of the Neurog2 mutant cortex and the Ascl1-null ventral telencephalon has been analysed to define genes downstream of both factors [53]. As with Hes1, there are striking overlaps in the sets of genes downstream of Ascl1, Neurog2 and Pax6 in the developing telencephalon.

**Figure 8:**
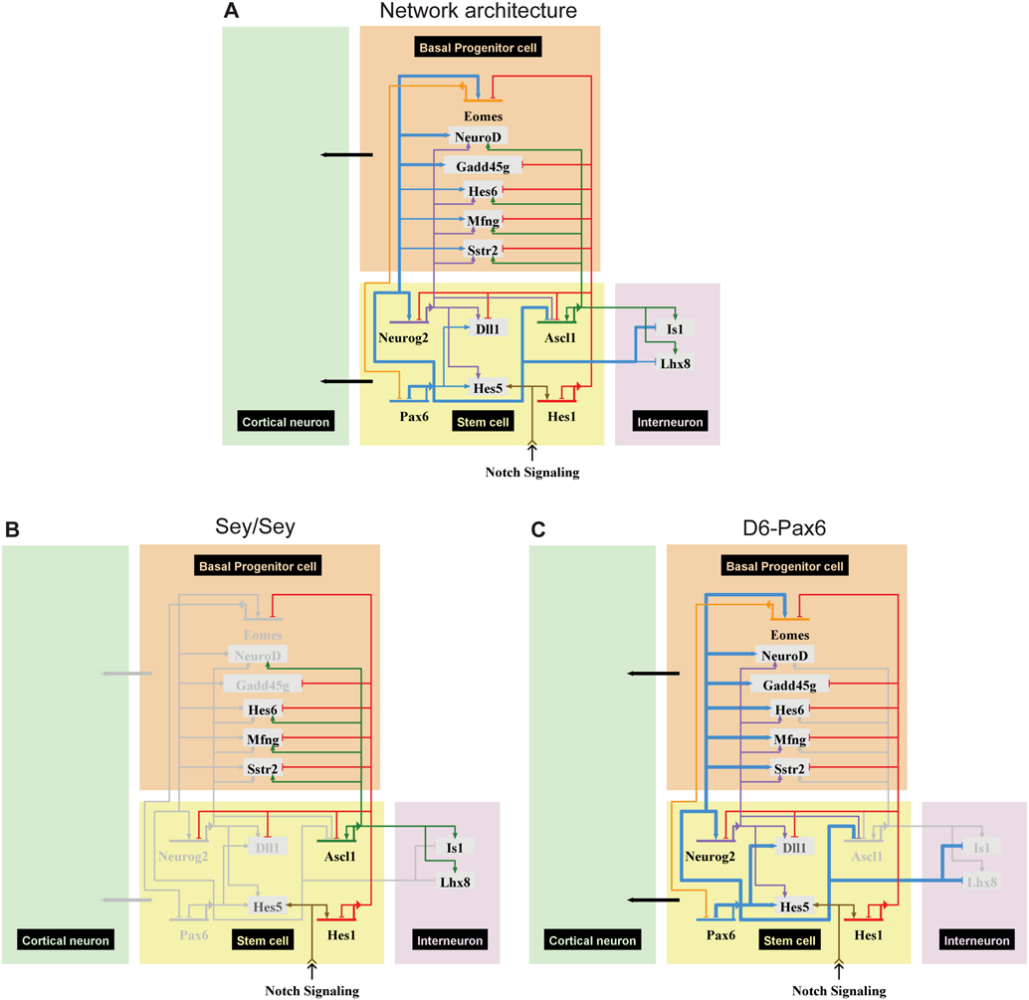
A neurogenesis and self-renewal regulatory circuit operating in neocortical stem cells, regulated by Pax6. (A) Pax6 binding and regulation data indicate that Pax6 levels are critical in maintaining the balance between stem cell maintenance, neurogenesis and SVZ genesis, as well as enforcing cortical identity. Combining these data with published data on genes downstream of Neurog2, Ascl1 and Hes1 (see text for details) enables construction of a basic network regulating cortical stem cell neurogenesis. Under normal conditions, Pax6 positively regulates Neurog2 and negatively regulates Ascl1 (Mash1), while directly and indirectly repressing the transcription factors Isl1 and Lhx8 respectively, both required for inhibitory interneuron genesis. Overall, this promotes genesis of glutamatergic projection neurons. Pax6 also positively regulates basal progenitor cell genes, including Eomes/Tbr2. Thick lines indicate direct regulation, thin lines indicate evidence from expression studies. (B) Loss of Pax6 function (Sey/Sey) removes the positive regulation of Neurog2 and the negative regulation of Ascl1, Lhx8 and Isl1. This would be predicted to result in the production of GABAergic interneurons in the cortex, as has been previously observed [52]. The loss of Pax6 also removes the positive regulation of the set of basal progenitor cell genes, however this is compensated for many of those genes in large part by the upregulation of Ascl1, with the exception of Eomes/Tbr2. Thus an SVZ is still generated in the absence of Pax6, but without Pax6 it loses its cortical identity and becomes Ascl1-expressing, similar to the SVZ of the ventral forebrain [14]. (C) Increasing the levels of Pax6 (indicated by thick lines) would be predicted to result in an over-production of basal progenitor cells by increasing Neurog2 expression and synergising with Neurog2 to increase expression of basal progenitor cell determinants, including Eomes/Tbr2. This increase in neurogenesis would potentially be at the expense of stem cell maintenance, although both Neurog2 and Pax6 increase expression of the stem cell maintenance factor Hes5.

Placing Pax6 in the context of these three transcription factors controlling cortical neurogenesis, Hes1, Ascl1 and Neurog2, enables the definition of a core transcriptional circuit controlling cortical neural stem cell self-renewal and neurogenesis (Figure 8A). Pax6 both positively regulates the expression of Neurog2 and also synergises with Neurog2 to promote basal progenitor cell genesis and thus production of cortical excitatory projection neurons, whereas Hes1 opposes this process by repressing many of the same genes. Ascl1 has complex functions in cortical neurogenesis: while it also promotes basal progenitor cell genesis, it also drives expression of transcription factors to promote inhibitory interneuron genesis (Lhx8 and Isl1, for example). Pax6 and Hes1 both repress Ascl1, and Pax6 represses Lhx8 and Isl1, to inhibit the interneuron-producing functions of Ascl1.

This network also leads to clear predictions of the normal functions of Pax6 in regulating neurogenesis and the consequences of altered Pax6 expression in the early cortex (Figure 8). The network indicates that Pax6 is essential for the cortical identity of the basal progenitor cells produced in the cortex, as it is an essential driver of Eomes/Tbr2 expression, a key determinant of cortical basal progenitor cell identity [47],[48]. Given the repression of Ascl1 by Pax6 in cortical stem cells and the interneuron-promoting function of Ascl1, loss of Pax6 function should result in a basal progenitor cell population lacking cortical identity (due to loss of Eomes/Tbr2 expression), a decrease in the genesis of cortical pyramidal neurons and an increase in the production of inhibitory interneurons (Figure 8B). These are all phenotypes observed in the Pax6 null cortex [8],[9],[14],[52].

In contrast, increasing Pax6 expression would be predicted to increase basal progenitor genesis early in cortical development and increase pyramidal cell genesis both directly from the ventricular zone and indirectly from basal progenitor cells (Figure 8C). In principle, Pax6 could increase cortical stem cell self-renewal/proliferation, but this would be in competition with all of the functions of Pax6 that promote neurogenesis: reduced proliferation/cell cycle exit (via Pten and Fzr1/Cdh1 expression), basal progenitor cell genesis (via Eomes/Tbr2 and Neurod1) and neurogenesis (via Neurog2 and Sox4). In the D6-Pax6 cortex, as described above, an increase in basal progenitor genesis is observed early in development, accompanied by an increase in the production of early-born cortical pyramidal cells.

### Levels of Pax6 Regulate the Balance between Neurogenesis and Self-Renewal

Our *in vivo* analysis supports the finding that Pax6 operates in and regulates a core transcriptional network that controls the balance between neurogenesis and stem cell self-renewal in a highly dosage-sensitive manner. Altering the amount of Pax6 in neural stem cells has profound effects on the output of neural stem cells, ultimately compromising the ability of neural stem cells to generate all of the required neurons for normal assembly of the cerebral cortex. This raises the question as to whether Pax6 levels do vary or oscillate *in vivo*, as has been reported for several other genes in developing systems [54].

**Figure 9:**
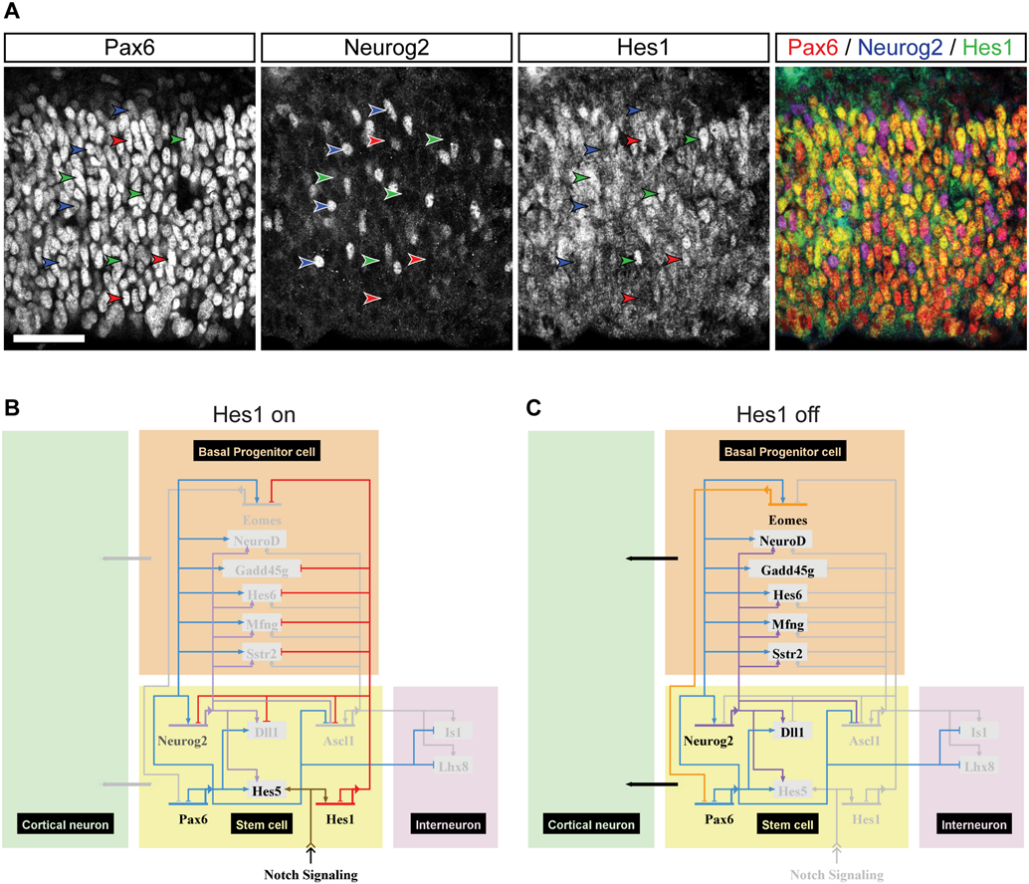
Pax6 protein levels are constant in cortical stem and progenitor cells, compared to the variation in Neurog2 and Hes1 levels: implications for network behaviour. (A) Confocal microscope images of immunofluorescent staining for Pax6, Neurog2 and Hes1 in the E12.5 cortex. Single channel images of staining for each antibody are shown, together with the merged image. Arrowheads highlight Pax6+ nuclei (red), Neurog2+ nuclei (blue) and Hes1+ nuclei (green). Almost all Hes1+ cells are also strongly Pax6+. Both Neurog2+/Pax6+ and Neurog2+/Pax6− cells were observed. Scale bar, 50 µm. (B) Prediction of the output of the self-renewal/neurogenesis network when Hes1 levels are high (active Notch signalling): under these conditions, Hes1 antagonises the positive regulation of Neurog2 by Pax6 to suppress neurogenesis. (C) Prediction of the output of the self-renewal/neurogenesis network when Hes1 levels are low (no active Notch signalling): under these conditions, Pax6 positively regulates Neurog2 expression, unopposed by Hes1. Pax6 and Neurog2 cooperate to promote neurogenesis and basal progenitor cell genesis.

The levels of Hes1 and Neurog2 proteins vary with cell cycle stage and have also been shown to oscillate in neocortical stem and progenitor cells with a 2–3 hour frequency [51], in a Notch-dependent fashion in the case of Hes1. Immunohistochemistry for Pax6, Neurog2 and Hes1 shows that Pax6 does not show obvious cell cycle-dependent changes in levels, unlike Hes1 and Neurog2, as assessed by nuclear location in the ventricular zone, as it is expressed in all neocortical stem and progenitor cells at relatively high levels (Figure 9A; Figure S2). This approach cannot resolve oscillations with a short periodicity, as observed for Hes1 and Neurog2 by live imaging [51]. However, Hes1-expressing cortical stem and progenitor cells also express high levels of Pax6, so it is unlikely that Hes1 represses Pax6 expression as it does Neurog2 [51]. In the presence of Notch signalling, increased Hes1 and Hes5 activity suppresses neurogenesis and promotes self-renewal, in part by repression of Neurog2, assisted by the activation of Hes5 expression by Pax6 (Figure 9B). In the absence of Notch signalling and Hes1 activity, Pax6-driven increase in Neurog2 and the resulting drive to neurogenesis are unopposed, allowing Pax6 to promote neurogenesis (Figure 9C).

Therefore, the identification of the direct transcriptional targets of Pax6 in neocortical stem and progenitor cells, combined with phenotypic analyses of the Pax6 gain and loss of function cortex, have enabled the elucidation of a regulatory network controlling the balance between neurogenesis and self-renewal. The development of this model provides a framework in which to explore questions arising from the mechanism reported here. For example, only a subset of Pax6+/Hes1− stem cells in G1 phase of the cell cycle go on to generate neurons in the next round of cell division. Therefore, there are likely to be additional components of this network that promote self-renewal and suppress neurogenesis.

## Discussion

Neural stem cell self-renewal, neurogenesis and cell fate determination are three forces that control the generation of specific classes of neurons at the correct place and time during development. Much remains to be discovered of the cellular networks operating to control these processes. We report here that the transcription factor Pax6 directly regulates genes controlling the balance between neocortical stem cell maintenance, neurogenesis and the production of basal progenitor cells in a dosage-dependent fashion. While consistent with genetic loss-of-function studies [10], [14], [20], [52], [55]–[58], the direct nature of the control of these processes by Pax6 and their dosage sensitivity are unexpected. We propose that this dosage sensitivity reflects the need for a critical level of Pax6 within neocortical stem and progenitor cells.

Using Pax6 ChIP, we have identified a set of the promoters bound by Pax6 in neocortical stem cells *in vivo* at E12.5. This set of Pax6-bound genes defines the components of potential networks regulated by Pax6 in this tissue, and is noteworthy for the enrichment for genes involved in controlling cell cycle progression, transcription factors expressed in the three main cell types found in the early cortex (ventricular zone stem cells, basal progenitor cells and differentiating neurons) and also transcription factors expressed specifically in non-cortical neurons, including the ventral forebrain, spinal cord and retina, as well as in pancreatic islet cells.

These binding data are compatible with a number of models for Pax6 action. For example, Pax6 could repress expression of genes that are not normally expressed in the neocortex, or alternatively could simply bind to target sites in the promoters of genes normally expressed in other cell types in a Pax6-dependent manner, without driving their expression in the cortex. Similarly, for those genes expressed in cortex, Pax6 could either positively or negatively regulate cell cycle progression, and positively or negatively regulate basal progenitor cell genesis. Evidence for gene regulation by Pax6 is essential to resolve these questions. Therefore, we combined the binding data with transcriptome data from Pax6 gain- and loss-of-function experiments in the early developing cortex in order to identify those genes whose expression is dependent on Pax6 and the nature of that dependency, finding that 22% of Pax6-bound genes show evidence for regulation *in vivo*.

Transcriptome analyses of Pax6 gain and loss of function cortices identified a subset of genes that show reciprocal regulation, but also clearly demonstrate that the gain and loss of function gene expression changes are not simply complementary, in agreement with the reported anatomical phenotypes of Pax6 null and Pax6 over-expressing cortices [14],[21],[27],[29]. However, there is a striking overlap and complementarity in the changes of expression in basal progenitor genes observed in each mutant. We found that Pax6 directly promotes expression of a large set of genes specifically expressed in basal progenitor cells, including the key determinant of that cell type, the transcription factor Eomes/Tbr2: mutations in Eomes/Tbr2 lead to a loss of the cortical intermediate progenitor cell population, accompanied by a reduction in neurons in all cortical layers [47],[48]. Increasing Pax6 levels drives basal progenitor cell genesis from cortical stem cells, primarily by increasing Eomes/Tbr2 expression, along with the other basal progenitor cell genes such as Gadd45g, Neurod1, Sstr2 and Hes6 [49]. Basal progenitor cells undergo a limited number of mitotic divisions to generate neurons [59], thus the overall effect of shifting the stem cell population towards basal progenitor cells is to increase neurogenesis at the expense of neural stem cell maintenance in the early stages of cortical development, ultimately resulting in microcephaly, as observed here.

However, there are also changes in expression specific to either gain or loss of Pax6 function, with many more genes showing altered expression upon increased Pax6 levels. For example, from the combined Pax6 binding and regulation data we have found that Pax6 expression positively regulates stem cell self-renewal by promoting expression of the transcription factor Hmga2 and the G1 cyclin dependent kinase, Cdk4. Hmga2 promotes neural stem cell self-renewal by reducing expression of two negative regulators of the cell cycle, p16^Ink4a^ and p19^Arf^ [39]. Hmga2 reduces expression of p16^Ink4a^ and p19^Arf^ indirectly via repression of JunB, a positive regulator of their expression, and we also found Pax6 binding to the promoter of JunB in cortical stem cells. p16^Ink4a^ slows cell cycle progression by inhibiting the G1 cyclin-dependent kinase Cdk4 [60], and Cdk4 is bound and positively-regulated by Pax6 in cortical stem cells. In contrast, Pax6 also directly promotes expression of Pten and Fzr1/Cdh1, both of which reduce neural stem cell proliferation and self-renewal [44],[61]. Thus, under normal conditions *in vivo* Pax6 has the potential to both promote and limit stem cell self-renewal. However, when Pax6 levels are increased, as in the D6-Pax6 transgenic cortex, the neurogenic functions of Pax6 are dominant over its ability to promote self-renewal.

We have also placed Pax6 in the context of other transcriptional regulators of self-renewal and neurogenesis, Hes1, Neurog2 and Ascl1/Mash1, in order to extend our coverage of the cellular networks controlling these processes. The marked overlap between those genes directly and indirectly regulated by Pax6 with the genes regulated by all three of the other factors provides strong evidence for the operation and architecture of the network regulating cortical neurogenesis, and the central importance of the basal progenitor population as a major output of that network. Pax6 and Neurog2 cooperate to promote neurogenesis, both directly and via the basal progenitor population, and this is opposed by the oscillating expression of Hes1 [51]. At the same time, Pax6 also promotes stem cell self-renewal in a manner that counterbalances its neurogenesis-promoting activity. However, when over-expressed, the promotion of neurogenesis and basal progenitor cell genesis by Pax6 is dominant over the promotion of self-renewal.

Therefore, we propose that there is an optimal level of Pax6 that determines the balance between neocortical stem cell self-renewal and neurogenesis: increasing that level drives stem cells to a neuronal or basal progenitor fate, whereas reducing the level leads to early cell cycle exit, manifest as increased early neurogenesis [14]. In both cases, altering Pax6 levels leads to a depletion of the stem cell population by exiting to neurogenesis, but by different pathways and with different neuronal fates: cortical pyramidal cells when Pax6 is increased, inhibitory interneurons when Pax6 is absent. The sensitivity of cortical development to Pax6 levels underlines the importance of assessing subtle structural and functional anomalies in humans heterozygous for Pax6 mutations, as has been done for aniridia patients [22],[25].

Finally, the findings of Pax6 function in neocortical stem and progenitor cells presented here have similarities with the functions of the ES cell pluripotency regulator Oct4 [3]. Oct4 shows marked dosage effects in ES cells *in vitro* such that a reduction in Oct4 levels leads to trophectoderm differentiation and a two-fold increase in Oct4 levels leads to differentiation to primitive endoderm and mesoderm [3]. Loss of Pax6 leads to a depletion of the cortical stem cell pool, via increased early neurogenesis secondary to a failure to self-renew, and also a switch in the fates of the neurons produced from glutamatergic cortical neurons to an inhibitory interneuron identity [52]. Similarly, increased Pax6 expression also leads to depletion of the stem cell pool, but in this case by driving stem cells to a basal progenitor fate, leading to an overproduction of early-born, deep-layer cortical neurons. Thus the level of Pax6 controls whether neural stem cells will self-renew, generate cortical neurons or produce basal progenitor cells.

## Materials and Methods

### Chromatin Immunoprecipitation

Chromatin immunoprecipitation (ChIP) was performed as described [62], with minor modifications. An average of 36 neocortices from E12.5 MF1 mouse embryos were used for each ChIP. For these and subsequent studies, all animal work was approved by local ethics review committees and, where relevant, carried out according to UK Home Office national guidelines. ChIP was carried out with specific rabbit polyclonal Pax6 antibodies generated against C-terminal peptides that recognise both splice variants of Pax6: C-term 1 -Chemicon, Cat no. AB5409 and C-term 2 - Covance PRB-278P. For array analysis, Pax6-bound genomic DNA was purified by ChIP using the Chemicon antibody, and ChIP material and whole cell extract DNA were globally amplified by ligation-mediated PCR [62]. To validate ChIP-on-chip data, unamplified ChIP material prepared with both Pax6 antibodies and from control samples lacking the primary antibody were used for gene-specific, quantitative PCR. Primers to amplify 100–250 bp target regions surrounding the predicted genomic binding locations for Pax6 were designed using Primer3 (http://frodo.wi.mit.edu/) [63] or Primer Express (Applied Biosystems), and checked for specificity in the genome using the BLAT algorithm (http://genome.ucsc.edu/). Quantitative PCR was carried out using the Roche Lightcycler system or the Applied Biosystems 7300 system. The amount of target regions was quantified in Pax6 and control chromatin immunoprecipitations lacking the primary antibody (no-antibody controls, NoAb). The enrichment for each gene was calculated by normalising the Pax6/NoAb ratio against the Pax6/NoAb ratio for a promoter region that was not found to be bound by Pax6 (non-bound control region from the Syt8 gene).

### Promoter Microarray Analysis

Amplified Pax6 ChIP and whole cell extract DNA were labelled by indirect incorporation of Cy3 or Cy5-labelled nucleotides and hybridised to Agilent mouse promoter 244 K arrays according to the manufacturer's instructions. Slides were scanned in an Agilent scanner and data extracted and normalised using Agilent Feature Extractor. Data were analysed by neighbour analysis [62] implemented in ChipAnalytics (Agilent) to calculate probability scores of Pax6 binding for each array oligonucleotide (p[Xbar] scores). Binding events were identified by sliding a 1000 bp window across the genomic space covered by the promoter array. Within a window, the best p[Xbar] score from each of the three ChIP-chip experiments was identified. A binding event was called if: (1) 2 or more scores were < = 0.015, and (2) the product of three scores was < = 0.000025. Gene ontology analysis of Pax6-bound genes was performed using GOToolBox (http://crfb.univ-mrs.fr/GOToolBox/home.php).

### D6-Pax6 Transgenic Mice

To generate D6-Pax6 transgenic mice, the 5.7 Kb D6 promoter fragment was cloned upstream of the canonical Pax6 open reading frame [35]–[37] and injected into the pronucleus of fertilized (C57BL/6×BALB/c) F1 mouse oocytes to generate founder mice. *In situ* RNA hybridization using digoxigenin (DIG)-labeled RNA probes was performed according to methods described at the Rubenstein lab website (http://physio.ucsf.edu/rubenstein/protocols/index.asp). Sections from the different genotypes (WT and D6-Pax6) were processed in parallel. Basal ganglia expression was used an internal control to compare results between different experiments and between experimental and WT samples. Probes used: *Pax6* (P. Gruss), *Neurog2* (F. Guillemot), *Tbr1* and *Eomes/Tbr2* (Rubenstein lab). Immunohistochemistry was performed on frozen sections (10 or 20 µm). Antibodies used: monoclonal anti-βIII-tubulin antibody (clone TUJ1; Covance), 1∶1000; anti-phospho-Histone H3 (Ser10) (Upstate) 1∶200; anti Pax6 (Developmental Studies Hybridoma Bank) 1∶1000; anti-Eomes/Tbr2 (gift from Robert Hevner, University of Washington School of Medicine, Seattle). Pax6 levels were quantified in 9 wildtype and 14 D6-Pax6 tissue sections by the intensity of the immunofluorescent signal: the signal along the dorsal-ventral axis was quantified in the ventricular zone by histogram (ImageJ, NIH), using the mean intensity. For this quantification the Developmental Studies Hybridoma Bank anti-Pax6 antibody was used at 1∶1000. TUNEL analysis was performed on 20 µm cryostat sections using the Apoptag Kit following the manufacturer's recommendations (Millipore, CA, USA). Similar results were observed with activated Caspase 3 staining (data not shown).

### Immunohistochemistry for Pax6, Neurog2, and Hes1

Triple immunohistochemistry for Pax6, Hes1 and Neurog2 was carried out on sections of E12.5 cortex by standard techniques (rabbit anti-Pax6 antibody, Covance PRB-278P; guinea pig anti-Hes1 antibody, gift from Ryoichiro Kageyama; goat anti-Neurog2, Santa Cruz Biotechnology). Quantification of the fluorescent intensity of nuclear staining for each antibody was carried out on confocal microscrope images (Radiance, Biorad) of a minimum of three sections using Volocity software (Improvision).

### mRNA Expression Profiling

Cortices were dissected from individual embryos in two litters of E12.5 Pax6^Sey^ (Edinburgh Small-eye, [6]) mutant embryos and from one litter of E12.5 D6-Pax6 transgenic mice. Total RNA was extracted and cDNA synthesised using the SMART system, as described [64]. Gene expression in neocortices from three single Pax6^Sey/Sey^ embryos was compared to that in four single wildtype littermates on six dye-swapped oligonucleotide microarrays; while gene expression in neocortices from three single D6-Pax6 embryos was compared to that in three single wildtype littermates at E12.5 in a set of 6 paired dye-swapped hybridizations. Arrays of the MEEBO oligonucleotide set (Invitrogen), produced by the Pathology Department, University of Cambridge were used for all studies. cDNA labeling, array hybridization, slide scanning (Axon GenePix microarray scanner, Molecular Devices) and data extraction were performed as described [64]. Expression data were archived and lowess normalized using the Acuity system (Molecular Devices). Log ratios of all expression measurements for each array were median-centered and expression ratios variance normalized across all of the arrays (without adjusting the average fold-change). Genes were filtered on the basis of the number of arrays on which they were detected, being required to be present in 4 of the 6 microarrays. For the D6-Pax6 analysis, dye-swapped replicate pairs of arrays were averaged, and in the case of one missing value, the single value used. Significant differences in gene expression were identified using the significance analysis of microarrays (SAM) algorithm (Version 2; one class, at least 200 permutations), using a false discovery rate (FDR) of 0.1 [65].

### Data Integration and Network Modelling

Dataset intersections between array datasets were performed using gene symbols. Gene symbols were retrieved using the SOURCE (http://smd-www.stanford.edu/cgi-bin/source/sourceSearch) database and the gene accession numbers provided by the array/oligonucleotide manufacturers. Network modeling was carried out using BioTapestry (www.biotapestry.org) [66].

## Supporting Information

Figure S1 Analysis of cell death in the Pax6-overexpressing cortex by TUNEL staining.
(10.00 MB TIF)

Figure S2 Quantification of Pax6 protein levels in individual cortical stem and progenitor cells.
(0.91 MB TIF)

Table S1 Gene promoters identified as bound by Pax6 by ChIP from E12.5 mouse cortex.
(0.36 MB XLS)

Table S2 Microarray analysis of altered mRNA levels in the E12.5 D6-Pax6 cortex, compared to wild-type littermates.
(0.82 MB XLS)

Table S3 Microarray analysis of altered mRNA levels in the E12.5 Sey/Sey cortex, compared to wild-type littermates.
(0.19 MB XLS)

Table S4 Sets of genes bound and regulated by Pax6 in the E12.5 cortex.
(0.05 MB XLS)

Table S5 Breakdown of gene expression patterns at E14.5 for genes bound and regulated by Pax6.
(0.03 MB XLS)
