# Supplementary figures and images for: The Level of the Transcription Factor Pax6 Is Essential for Controlling the Balance between Neural Stem Cell Self-Renewal and Neurogenesis
Source: PLoS Genet. 2009 Jun 12;5(6):e1000511. doi: 10.1371/journal.pgen.1000511 (PMC2686252; doi:10.1371/journal.pgen.1000511)

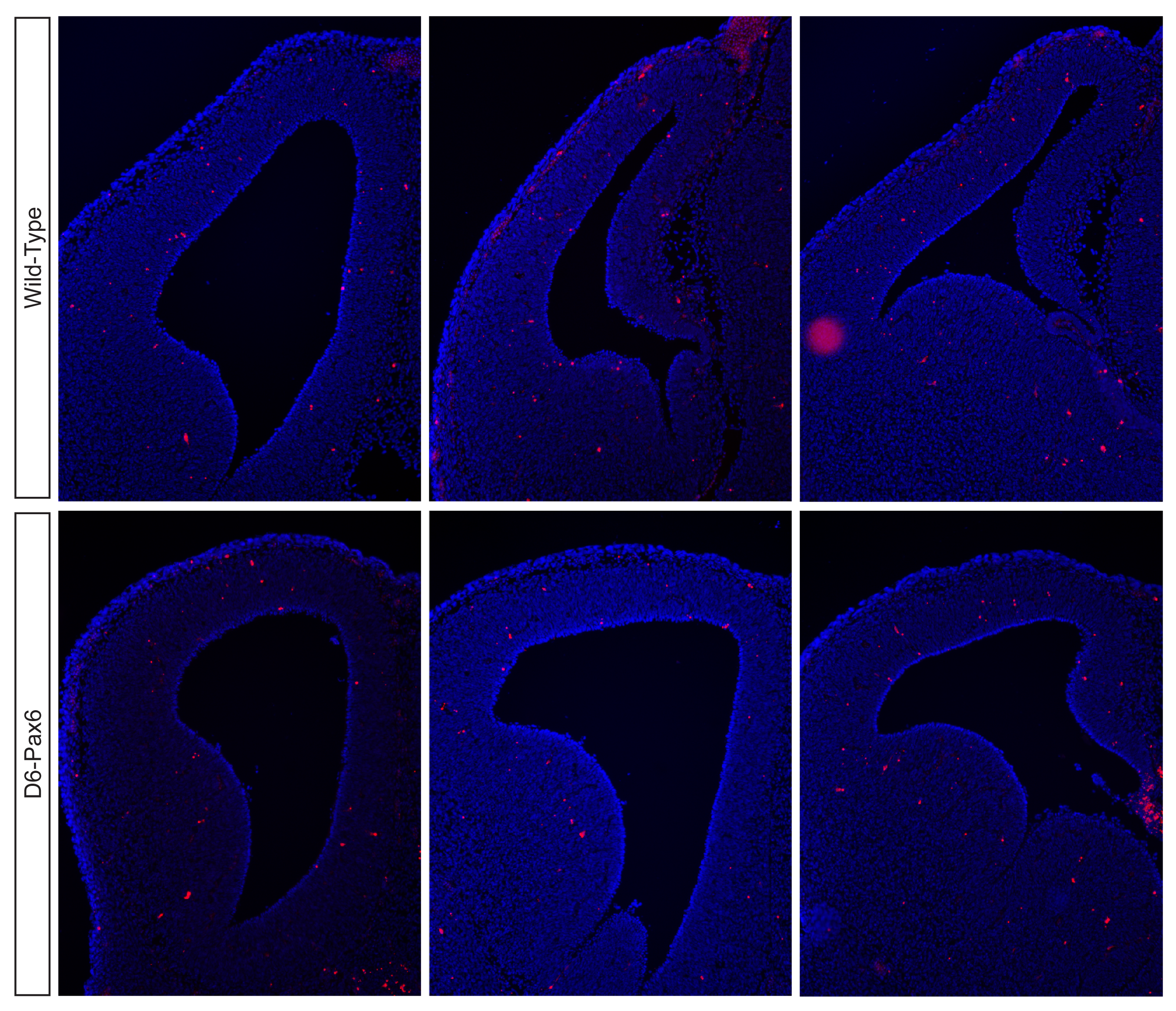

Supplement: Figure S1 — Analysis of cell death in the Pax6-overexpressing cortex by TUNEL staining. (10.00 MB TIF) [file pgen.1000511.s001.tif]

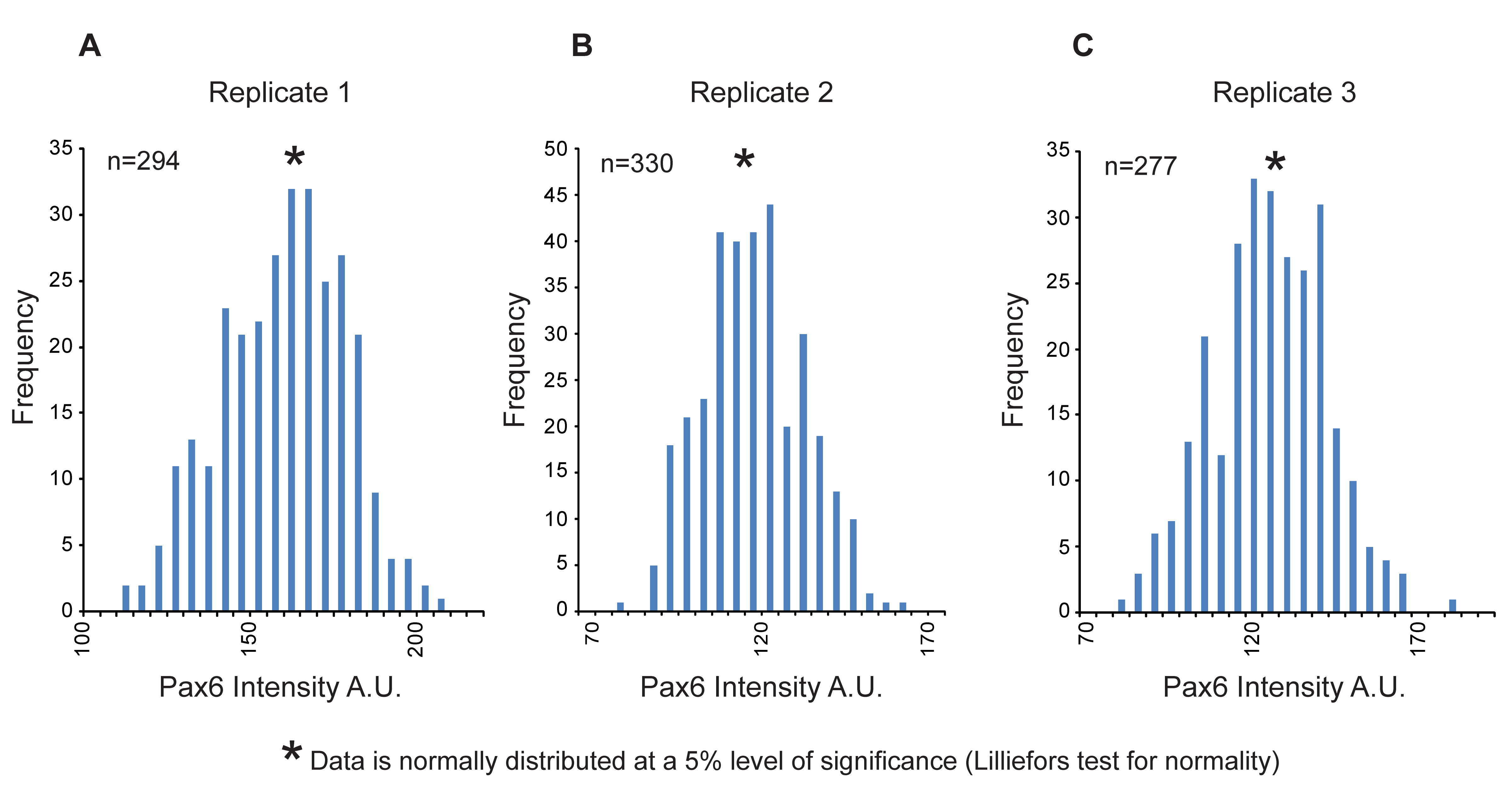

Supplement: Figure S2 — Quantification of Pax6 protein levels in individual cortical stem and progenitor cells. (0.91 MB TIF) [file pgen.1000511.s002.tif]
